# Crack Sealing in Concrete with Biogrout: Sustainable Approach to Enhancing Mechanical Strength and Water Resistance

**DOI:** 10.3390/ma17246283

**Published:** 2024-12-23

**Authors:** Jian Wang, Shengjie Ji, Shuguang Huang, Zihang Jiang, Siqi Wang, Huaiqi Zhang, Zijian Wang, Junfei Zhang

**Affiliations:** 1 Beijing Building Research Institute Corporation Ltd., China State Construction Engineering Corporation, Beijing 100076, Chinazihangjiang@163.com (Z.J.); wangsiqi@163.com (S.W.); 2 Institute of Acoustics, Chinese Academy of Sciences, Beijing 100190, China; 3 School of Civil and Transportation Engineering, Hebei University of Technology, Tianjin 300401, Chinazijianwang@hebut.edu.cn (Z.W.)

**Keywords:** microbial-induced carbonate precipitation (MICP), biogrout, concrete, cracking, mechanical properties, water resistance

## Abstract

Concrete, as the most widely used construction material globally, is prone to cracking under the influence of external factors such as mechanical loads, temperature fluctuations, chemical corrosion, and freeze–thaw cycles. Traditional concrete crack repair methods, such as epoxy resins and polymer mortars, often suffer from a limited permeability, poor compatibility with substrates, and insufficient long-term durability. Microbial biogrouting technology, leveraging microbial-induced calcium carbonate precipitation (MICP), has emerged as a promising alternative for crack sealing. This study aimed to explore the potential of *Bacillus pasteurii* for repairing concrete cracks to enhance compressive strength and permeability performance post-repair. Experiments were conducted to evaluate the bacterial growth cycle and urease activity under varying concentrations of Ca^2+^. The results indicated that the optimal time for crack repair occurred 24–36 h after bacterial cultivation. Additionally, the study revealed an inhibitory effect of high calcium ion concentrations on urease activity, with the optimal concentration identified as 1 mol/L. Compressive strength and water absorption tests were performed on repaired concrete specimens. The compressive strength of specimens with cracks of varying dimensions improved by 4.01–11.4% post-repair, with the highest improvement observed for specimens with 1 mm wide and 10 mm deep cracks, reaching an increase of 11.4%. In the water absorption tests conducted over 24 h, the average mass water absorption rate decreased by 31.36% for specimens with 0.5 mm cracks, 29.06% for 1 mm cracks, 27.9% for 2 mm cracks, and 28.2% for 3 mm cracks. X-ray diffraction (XRD) and scanning electron microscopy (SEM) analyses confirmed the formation of dense calcium carbonate precipitates, with the SEM–EDS results identifying calcite and vaterite as the predominant self-healing products. This study underscores the potential of MICP-based microbial biogrouting as a sustainable and effective solution for enhancing the mechanical and durability properties of repaired concrete.

## 1. Introduction

The repair of concrete cracks is a critical issue in civil and hydraulic engineering, as it directly impacts the durability and safety of structures. Traditional crack repair techniques, such as epoxy resins and polymer mortars, offer some level of crack sealing, but are limited by issues related to material permeability and a poor compatibility with cement-based materials [1,2,3]. Due to their high viscosity, these materials often fail to penetrate shallow or fine cracks. Additionally, traditional materials are prone to aging and deformation with extended use, diminishing the repair effectiveness and durability of structures [4,5,6]. These shortcomings highlight the significant engineering value and theoretical importance of developing new and effective concrete crack repair technologies.

In recent years, microbial biogrouting has garnered significant attention as an innovative crack repair method. This technique utilizes the microbial-induced mineralization process to precipitate calcium carbonate within cracks. By using its unique negative-pressure permeation properties, microbial biogrouting can deeply penetrate cracks, achieve effective sealing, and even exhibit self-healing capabilities in response to dynamic crack changes [4,7,8]. Compared to traditional methods, microbial-induced calcium carbonate precipitation (MICP) technology addresses issues such as inadequate permeability and environmental concerns, offering a more sustainable and efficient alternative. Its advantages include a low viscosity, allowing for penetration into fine cracks, autonomous crack localization and sealing without excessive external intervention, and providing an efficient, durable, and environmentally friendly solution for crack repair [9,10,11].

**Figure 1:**
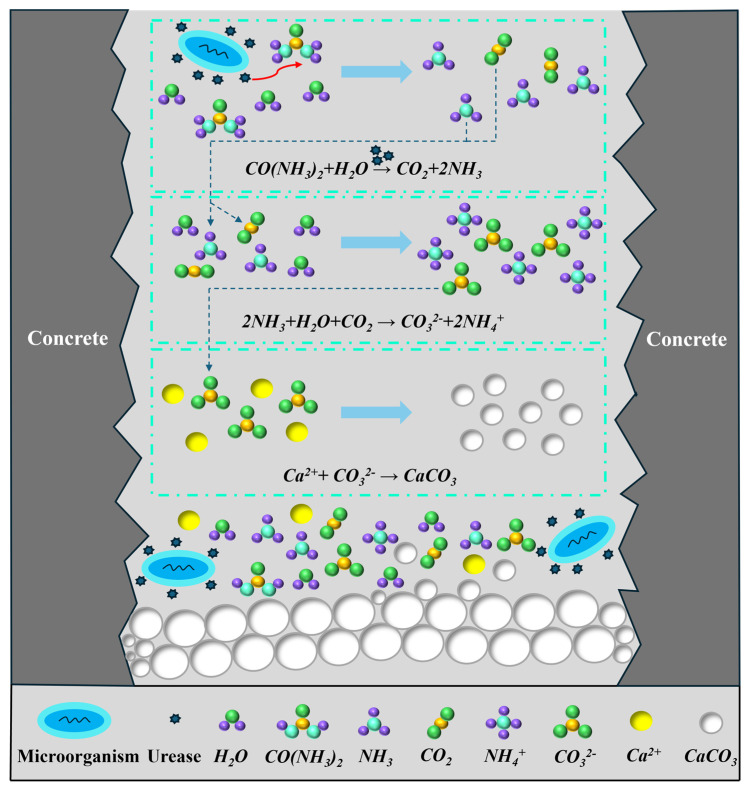
Schematic diagram of MICP repairing concrete cracks.

MICP achieves these results through the three following primary pathways: urea hydrolysis, denitrification, and organic matter degradation, with the urea hydrolysis pathway being the most commonly applied and widely studied. Among the microorganisms used in this pathway, *Bacillus pasteurii* is the most prevalent. The mechanism of the MICP process, as illustrated in Figure 1, can be divided into three distinct stages. In the first stage, microbes rapidly proliferate and produce urease. Under the influence of these microbes, urease catalyzes the decomposition of urea and water, resulting in the formation of NH_3_ and CO_2_. In the second stage, the CO_2_ reacts with water and the NH_3_ produced in the solution, ultimately forming NH_4_^+^ and CO_3_^2−^. In the third stage, the positively charged Ca^2+^ ions are attracted to the negatively charged bacterial surface and react with ${CO}_{3}^{2-}$, leading to mineralization and the eventual precipitation of CaCO_3_ [12,13,14,15]. Calcium carbonate gradually forms, filling the voids in the concrete cracks and “bridging” the concrete on either side of the crack. This bridging effect helps to enhance the load-bearing and deformation capacities of the cracked region [16,17]. Over the years, extensive research has been conducted on the calcium carbonate precipitation process induced by *Bacillus pasteurii*. Xu et al. [18] demonstrated through compressive strength tests that mineralization repair using *Bacillus pasteurii* significantly improved the strength of repaired concrete specimens. Their experimental results showed that a 0.41 mm wide crack was completely filled. The compressive strength of the repaired specimens reached 39.3 MPa, which was 2.3 times the pre-repair strength of 16.8 MPa. Jia et al. [10] further validated this finding through water permeability tests, showing that the permeability of concrete specimens improved with increasing *Bacillus pasteurii* inoculation cycles. After 12 cycles, the water flow rate under a 500 mm hydraulic head was reduced to 0 mL/min. Nosouhian et al. [19] demonstrated through rapid chloride ion penetration and permeability tests that mineral deposits produced by *Bacillus pasteurii* improved the impermeability of concrete specimens. Their results indicated a 12.15% reduction in the average charge passed and a 12.14% decrease in water absorption after repair.

Although significant progress has been made in using MICP for concrete crack repair, showcasing its immense potential for enhancing the mechanical properties and durability of cracked concrete, most existing studies focus primarily on the experimental validation of MICP’s crack-sealing efficacy. Research on critical influencing factors, such as crack dimensions and characteristics, remains limited. Factors such as crack width and depth play crucial roles in determining the repair efficiency of MICP. Therefore, the performance testing of specimens with cracks of varying sizes is essential to elucidate the intrinsic relationship between crack morphology and post-repair mechanical and transport properties.

This study aimed to utilize *Bacillus pasteurii* for crack repair to enhance the compressive strength and permeability performance of repaired concrete. The optimal mineralization time and calcium ion concentration for *Bacillus pasteurii* were determined by analyzing its growth cycle characteristics and testing the ideal calcium ion concentration for inducing mineralization. Standard cement mortar specimens with pre-fabricated cracks were used as repair targets to evaluate the effectiveness of microbial grouting-induced mineralization technology for crack sealing and repair. Experimental analyses were conducted to investigate the mechanisms of microbial-induced mineralization in crack repair and the deposition characteristics of mineralized products within the crack regions. Compressive strength tests and water absorption tests were performed before and after microbial repair to compare the changes in compressive strength and mass water absorption rates. This analysis explored the intrinsic relationship between crack morphology and transport properties. X-ray diffraction (XRD) and scanning electron microscopy (SEM) were employed to characterize the microstructures of the mineralized products and the sealing interfaces. These techniques revealed the crystalline phases and distribution patterns of the mineralized products within the repaired crack regions. The findings of this study further validate the potential of MICP as a scalable, multifunctional, and sustainable solution for infrastructure repair. 

## 2. Materials and Methods

### 2.1. Raw Materials

The main materials used in the microbial experiments included *Bacillus pasteurii* (North Bio Company, strain ATCC 11859; Gram-positive; rod-shaped cells, 2–3 µm in length; spherical spores, 0.5–1.5 µm in length), beef extract, urea, peptone, calcium acetate, and deionized water. All reagents used in the experiments were sourced from the China National Pharmaceutical Group Chemical Reagents Platform. The composition of the bacterial enrichment medium is shown in Table 1. Ordinary Portland cement (OPC) with CEM I 42.5 R was used in this study. The chemical composition of OPC is shown in Table 2. The sand used was standard sand produced by Xiamen ISO Standard Sand Co., Ltd. (Xiamen, China).

### 2.2. Growth Assessment and Urease Activity Measurement of Bacillus pasteurii

In this study, the growth of *Bacillus pasteurii* was characterized using the optical density method. The bacterial growth was assessed by measuring the optical density of the bacterial suspension at a wavelength (λ) of 600 nm using a UV—visible spectrophotometer [20,21]. Initially, 250 mL of a uniformly prepared liquid medium was placed in a conical flask and sealed with a breathable membrane. The flask was then autoclaved at 121 °C with high-pressure steam for 25 min and allowed to cool to room temperature. Under aseptic conditions on a clean bench, 10 mL of bacterial culture was inoculated into the liquid medium. After inoculation, the culture was incubated on a constant-temperature orbital shaker. At designated time points, 2 mL of the culture was sampled and placed in a cuvette to measure its optical density at 600 nm.

The level of urease activity affects the rate of urea hydrolysis, which, in turn, influences the rate and efficiency of microbial mineralization reactions. In the process of mineralization repair, the most significant factor affecting urease activity is the concentration of Ca^2+^ in the solution [22,23]. Therefore, it is necessary to measure the changes in urease activity under different calcium ion concentrations to select the most suitable concentration of calcium acetate. The optimization of urease activity under varying calcium ion concentrations was primarily investigated by quantifying the amount of urea hydrolyzed, as urease activity can be represented by the rate of urea decomposition. During the hydrolysis of urea, urease-producing bacteria generate NH_4_^+^ and CO_3_^2−^, which increase the solution’s electrical conductivity. Studies have demonstrated a linear relationship between solution conductivity and the amount of urea decomposed. Therefore, the change in conductivity over time, measured using a conductivity meter, serves as an indirect indicator of urease activity. Since the rate of urea hydrolysis by urease inevitably varies with different calcium ion concentrations, the conductivity of the solution will also differ under identical reaction times. By comparing the conductivity of solutions with varying calcium ion concentrations but a consistent urea concentration, the urease activity can be evaluated. This method enables the identification of the optimal calcium ion concentration to maximize the urease activity of *Bacillus pasteurii*. Urease activity (U) is calculated using Equation (1) [24], as follows:

(1) $$U=\frac{∆T}{t}\times \frac{1000}{60}$$

where ∆T is the change in solution conductivity within 5 min, ms, and t is the detection time, 5 min.

### 2.3. Specimen Preparation

During the repair of concrete cracks, the width, depth, and length of the cracks are critical factors that must be considered. In this study, the crack length was set at 20 mm, and the effects of microbial repair on different cracks were investigated by controlling the following two variables: crack width and depth. Cubic standard cement mortar specimens with an edge length of 40 mm were prepared, with a material quality ratio of water–cement–standard sand = 1:2:6. Concrete cracks were created using a pre-set steel plate method, where a bridge-type steel plate coated with lubricant was fixed in the middle of the mold during concrete casting. After casting for 3 h, the bridge-type steel piece was slowly pulled vertically but not removed. After 6 h of casting, the bridge-type steel piece was removed from the concrete specimen. In this study, concrete pre-set cracks with widths of 0.5 mm, 1 mm, 2 mm, 3 mm and depths of 10 mm, 20 mm, and 30 mm were created. The scheme was designed as shown in Table 3. After curing the specimens in a standard curing room for 28 days, the specimens were cleaned to ensure that the pre-fabricated cracks were intact, clean, and free of impurities and loose materials. Following natural drying, microbial remediation experiments were conducted.

**Figure 2:**
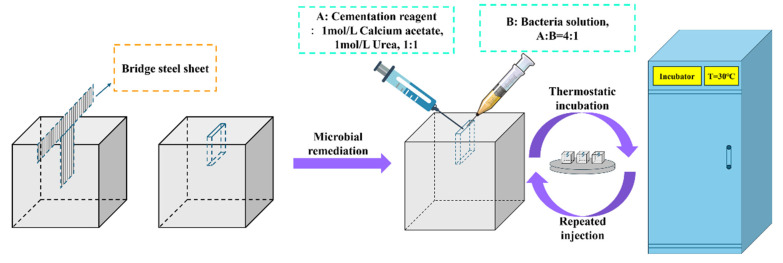
The experimental process.

In the microbial remediation experiments, the composition of the binding solution consisted of 1 mol/L of calcium acetate and 1 mol/L of urea solution, with a composite ratio of 1:1. The ratio of bacterial solution to binding solution was 1:4. In the experiment, the bacterial solution used was prepared by diluting the original bacterial sludge, obtained after high-speed centrifugation, with nutrients. The experiment employed an injection method using specialized injection equipment to simultaneously inject the bacterial and binding solutions into the cracks, controlling the injection speed and pressure to ensure a uniform distribution of the mixture within the cracks. After one injection, the specimen was placed in a constant-temperature (T = 30 °C) incubator and left undisturbed. Every 12 h, the bacterial and binding solutions were injected again, with a total of 60 injections. The experimental process is illustrated in Figure 2.

### 2.4. Methods for Evaluating the Effectiveness of Microbial Remediation of Cracks

#### 2.4.1. Compressive Strength Test

Following the microbial repair of the specimen cracks, compressive strength tests were conducted using either a hydraulic press or a universal testing machine equipped with appropriate load sensors. Regular calibration of the equipment was performed to ensure accuracy. During the experiments, the side of the specimen with the crack was positioned laterally. Furthermore, it was ensured that the load was uniformly applied across the entire cross-sectional area of the specimen. The compressive strength tests were carried out at a constant loading rate of 2400 N/s, gradually increasing the load until the specimen failed. The maximum load at failure was recorded. For each group of specimens with different crack sizes, three sets of cement mortar samples were tested, and the average strength value of the three samples was recorded as the strength value for that specific group.

#### 2.4.2. Water Absorption Experiment

**Figure 3:**
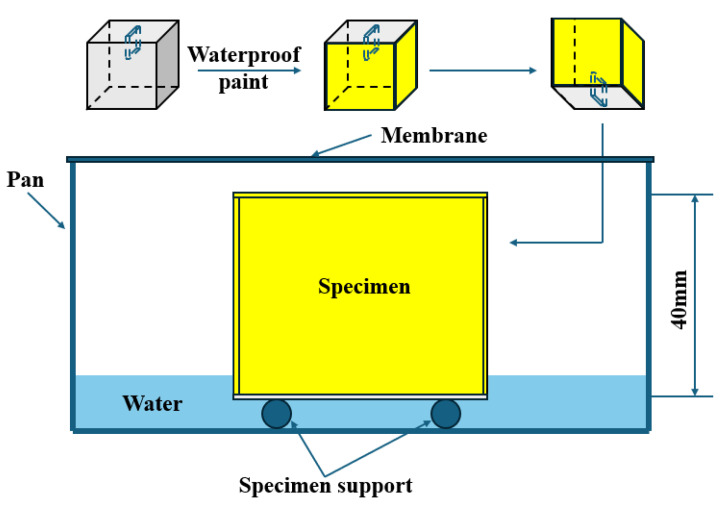
The process of the water absorption experiment.

Water absorption experiments were conducted by measuring the mass change in the concrete specimens before and after immersion. Initially, the specimens were placed in a desiccator within an oven set at 50 ± 2 °C for 24 h. Subsequently, the surfaces of the concrete specimens (excluding the cracked surfaces) were uniformly coated with a waterproof paint to a certain thickness. After application, the waterproof coating was allowed to cure for 24 h. Each specimen’s mass was measured with a precision of 0.01 g. A support device was placed at the bottom of a container, which was then filled with tap water such that the water level was 3 to 5 mm above the top of the support. During the experiment, the water level was maintained at 1 to 3 mm above the top of the support device. At predetermined time intervals of 4, 8, 12, 24, 36, and 48 h, the specimens were removed from the container, and any surface water was absorbed with a damp paper towel or cloth. After removing excess moisture, the specimens were inverted to ensure that the wet surfaces did not contact the balance pan (which must be dry), and the mass difference before and after immersion was calculated [25]. The process of the water absorption experiment is illustrated in Figure 3.

#### 2.4.3. Composition and Microstructure Analysis

The test materials were subjected to X-ray diffraction (XRD) analysis using the SmartLab SE X-ray diffractometer manufactured by Rigaku Corporation, Akishima-shi, Japan, to analyze their phase composition and crystal structure. The test target employed was a copper target, with a selected scanning range from 10 to 80° and a scanning speed of 5°/min. 

The microstructures were captured using an S4800 scanning electron microscope (SEM) manufactured by Hitachi, Tokyo, Japan. This instrument has a maximum resolution of 1.4 nm, magnification ranging from 20 to 800,000 times, and an acceleration voltage in the range from 0.5 to 30 kV.

## 3. Results 

### 3.1. Growth Conditions of Bacillus pasteurii and Optimal Ca^2+^ Concentration

**Figure 4:**
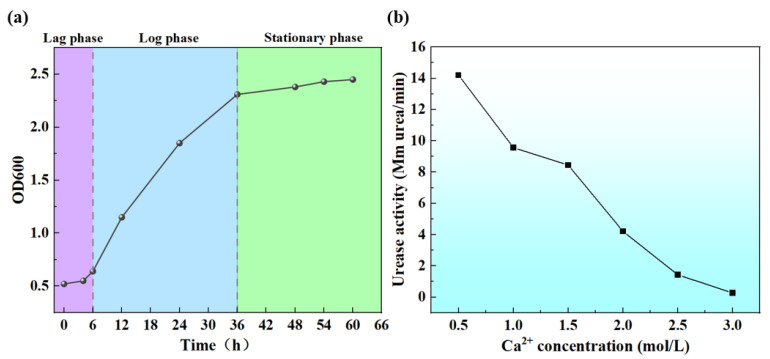
Growth conditions of *Bacillus pasteurii* and urease activity assay: (**a**) the growth of *Bacillus pasteurii* within 60 h and (**b**) the variation in urease activity with changes in Ca^2+^.

The growth of *Bacillus pasteurii* within 60 h is illustrated in Table 4 and Figure 4a. As can be seen, from 0 to approximately 6 h, the OD600 increased from 0.52 to 0.64, indicating that the microorganisms were adapting to the environment and increasing their metabolic and biochemical activities but had not yet significant reproduction, hence, they were in the lag phase. From approximately 6 to 36 h, the OD600 increased significantly from 0.64 to 2.31, demonstrating that the microorganisms were rapidly dividing and proliferating during this period, and were in the logarithmic phase. During this stage, the number of microorganisms grew exponentially, and typically, this is considered to be the optimal period for studying microbial physiological characteristics, as the metabolic activity of the cells is highest [26]. Beginning at 36 h, the growth rate of OD600 slowed, reaching 2.45 by 60 h, suggesting that the growth of the microorganisms had essentially stabilized, with the rate of new cell generation being roughly equal to the cell death rate, thereby indicating a stationary phase. When utilizing cultured *Bacillus pasteurii* for mineralization repair, it is ideal to perform this during a period of active bacterial growth and when cell numbers are high, to ensure that sufficient bacteria are available for the calcium deposition process. Therefore, crack repair should be conducted during the late logarithmic to early stationary phase (from approximately 24 to 36 h).

The effectiveness of *Bacillus pasteurii* in repairing concrete cracks is closely related to its growth behavior and the urease activity produced, as the growth of the bacteria directly influences its bioactivity. Only actively growing bacteria can effectively perform biomineralization. Furthermore, the activity of urease significantly influences both the rate and quantity of calcium carbonate formation, thereby affecting the speed and outcome of crack repair. This is because urease catalyzes the hydrolysis of urea, producing ammonia (NH_3_) and carbon dioxide (CO_2_), which subsequently react to form carbonate ions (CO_3_^2−^). These carbonate ions combine with calcium ions (Ca^2+^) to precipitate as calcium carbonate (CaCO_3_). A higher urease activity leads to faster urea hydrolysis, increasing the rate of carbonate ion production and thereby accelerating calcium carbonate precipitation. This acceleration significantly influences the timing of precipitation onset and the efficiency of crack filling. Furthermore, urease activity affects the spatial distribution of calcium carbonate precipitation within cracks. When urease activity is high, the resulting calcium carbonate precipitates more rapidly and diffuses efficiently within the crack, ensuring a broader and more uniform coverage. However, an excessively high urease activity may result in localized over-deposition, potentially causing uneven filling in deeper or wider regions of the crack. Conversely, a low urease activity may lead to insufficient precipitation, making it challenging to effectively seal cracks. As illustrated in Figure 4b, the Ca^2+^ concentration has a significant inhibitory effect on urease activity. Moreover, the higher the Ca^2+^ concentration, the more pronounced the inhibition of urease activity [22,27]. The figure indicates that, when the Ca^2+^ concentration is 0.5 mol/L, urease activity reaches its peak at 14.2 mM urea/min. However, when this concentration increases to 3 mol/L, urease activity drops to only 0.27 mM urea/min. This is because the cell membrane regulates the exchange of materials between the intracellular and extracellular environments. When high concentrations of Ca^2+^ are present in the solution, Ca^2+^ is adsorbed onto the cell surface, reducing the material exchange pathways of the cell membrane and making it difficult for urease to migrate, thus decreasing its activity [28]. However, if the Ca^2+^ concentration is too low, it affects the formation of calcium carbonate precipitation. Therefore, 1 mol/L of calcium acetate was chosen as a component of the cementing fluid.

### 3.2. Compressive Strength

**Figure 5:**
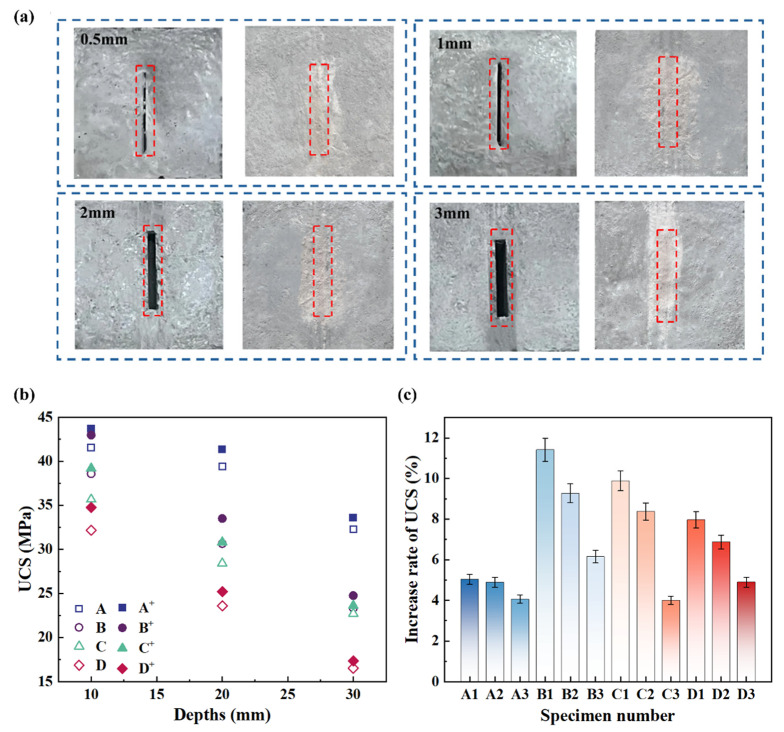
Visualization and UCS variations in repaired concrete cracks: (**a**) comparison of mineralization repair for cracks of different widths before and after treatment, (**b**) compressive strength before and after crack repair, and (**c**) increase ratio in compressive strength after repair.

A comparison of the mineralization repair of cracks with different widths before and after treatment is shown in Figure 5a. 

Figure 5a illustrates a visual comparison of crack repair using MICP across concrete specimens with varying crack widths. The two images in each group display the condition of the cracks before and after the MICP treatment, with the red dashed boxes highlighting the regions of interest. In all cases, it is evident that MICP effectively contributed to the partial or full closure of cracks through the bio-mineralization process. During this process, the bacterial-induced precipitation of CaCO_3_ filled the crack voids, leading to observable differences in crack visibility pre- and post-treatment.

The results presented in Table 5 and Figure 5b,c show the uniaxial compressive strength (UCS) of the cracked concrete specimens before and after MICP treatment. It was found that specimens with cracks with a 0.5 mm width (Group A) exhibited a modest strength increase between 4.06% and 5.04%, with deeper cracks showing slightly lower gains. In contrast, specimens with cracks with a 1 mm width (Group B) showed the most significant increase in UCS, with improvements ranging from 6.15% to 11.4%, with the shallowest crack (B_1_) achieving the highest strength gain. Noticeably, specimens with the widest cracks (3 mm) had the smallest improvements in strength, ranging from 4.90% to 7.97% [29,30].

### 3.3. Water Absorption Experiment

**Figure 6:**
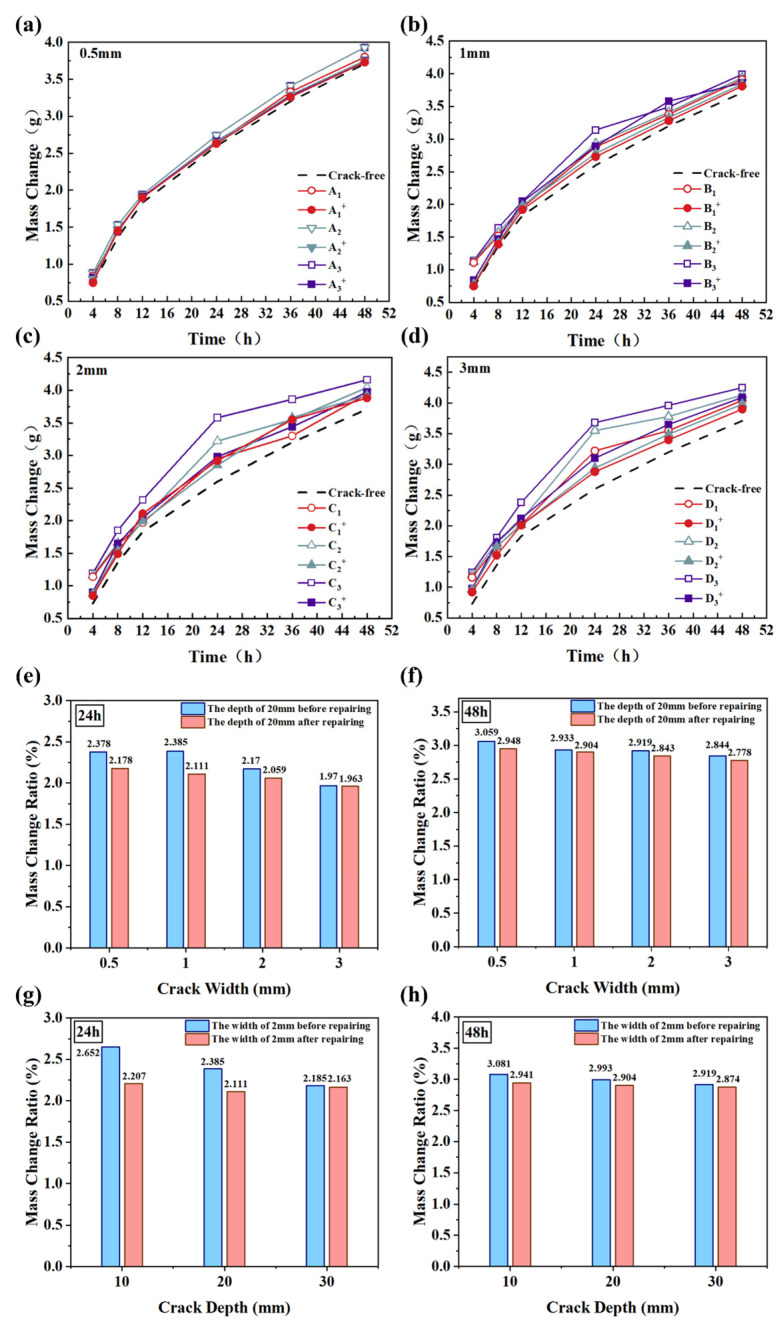
Water absorption and rate of change in water absorption of different crack specimens over time: (**a**–**d**) the variation in water absorption over time and crack depth for crack widths of 0.5 mm, 1 mm, 2 mm, and 3 mm before and after repair, (**e**,**f**) the water absorption rates of cracks with a width of 2 mm at different depths after 24 h and 48 h, and (**g**,**h**) the water absorption rates of cracks with a width of 2 mm at different depths after 24 h and 48 h.

Figure 6a–d illustrate the trends in the capillary water absorption of the crack concrete specimens with varying crack parameters over time. Figure 6e–h also present a comparison of the absorbed water mass change ratios of different groups at 24 h and 48 h when fixing the crack depth and width, respectively. The data in Figure 6a–d clearly show that the capillary water absorption for all specimens increased gradually, with a rapid increase from 0 to 24 h, after which the rate of increase slowed. In addition, it was found that the biogrout for concrete crack repair was significantly effective, particularly when the depth and width of the crack were relatively small. For instance, when the crack depth was held constant at 20 mm, the mass change ratio of the absorbed water after 24 h was reduced significantly following bio-mineralization. As observed from the 24-h water absorption test, the specimens with crack widths of 0.5 mm, 1 mm, 2 mm, and 3 mm exhibited reductions in water absorption mass of 0.03 g, 0.18 g, 0.33 g, and 0.34 g, respectively, after crack repair. Correspondingly, the average water absorption rates decreased by 31.36%, 29.06%, 27.9%, and 28.2%, respectively. In Figure 6e, the absorbed water mass change exhibits a pronounced decrease when the crack width is 0.5 mm and 1 mm, respectively. Similarly, when the crack width was fixed at 2 mm, the mass change ratio of the absorbed water after 24 h was reduced significantly after bio-mineralization for a crack depth of 10 mm. The depth and width of cracks influence the efficacy of the MICP process, with smaller cracks exhibiting better sealing due to increased CaCO_3_ dep please revise the sentence “enhancing their physiological activities” to make readers to better undestand the meaning based on the context of the sentences” Figure 6a–d illustrate the trends in the capillary water absorption of the crack con-crete specimens with varying crack parameters over time. Figure 6e–h also present a comparison of the absorbed water mass change ratios of different groups at 24 h and 48 h when fixing the crack depth and width, respectively. The data in Figure 6a–d clearly show that the capillary water absorption for all specimens increased gradually, with a rapid increase from 0 to 24 h, after which the rate of increase slowed. In addition, it was found that the biogrout for concrete crack repair was significantly effective, particularly when the depth and widthof the crack were relatively small. For instance, when the crack depth was held constant at 20 mm, the mass change ratio of the absorbed water after 24 h was reduced significantly following bio-mineralization. As observed from the 24-h water absorption test, the specimens with crack widths of 0.5 mm, 1 mm, 2 mm, and 3 mm exhibited reductions in water absorption mass of 0.03 g, 0.18 g, 0.33 g, and 0.34 g, respectively, after crack repair. Correspondingly, the average water absorption rates de-creased by 31.36%, 29.06%, 27.9%, and 28.2%, respectively. In Figure 6e, the absorbed wa-ter mass change exhibits a pronounced decrease when the crack width is 0.5 mm and 1 mm, respectively. Similarly, when the crack width was fixed at 2 mm, the mass change ratio of the absorbed water after 24 h was reduced significantly after bio-mineralization for a crack depth of 10 mm. The depth and width of cracks influence the efficacy of the MICP process, with smaller cracks exhibiting better sealing due to increased CaCO_3_ deposition [31]. Notably, it was also observed that there was no significant difference in the absorbed water mass change ratio when measuring it after 48 h. This phenomenon suggests that saturation equilibrium was reached within the concrete pores even when the crack was sealed by CaCO_3_ precipitates “osition [31]. Notably, it was also observed that there was no significant difference in the absorbed water mass change ratio when measuring it after 48 h. This phenomenon suggests that even after the cracks were sealed by CaCO_3_ precipitates, the water absorption process reached a point of equilibrium within the concrete pores [32].

Narrower cracks (e.g., 0.5 mm and 1 mm) exhibited stronger capillary action compared to wider cracks. This enhanced capillary effect facilitated the infiltration and distribution of microbial suspensions and binding solutions within the crack network, ensuring a more uniform calcium carbonate precipitation (CaCO_3_ throughout the crack. According to principles of fluid mechanics, narrower channels amplify capillary-driven flow, increasing the retention time of the solution and enhancing the overall reaction efficiency [21]. Additionally, the spatial constraints of narrower cracks create an environment conducive to higher local reactant concentrations. This limited volume restricts the diffusion of reactants, promoting their interaction within a smaller area, thereby improving precipitation efficiency and the effectiveness of crack sealing. In contrast, wider cracks result in diluted microbial solutions, leading to a reduced local activity and diminished repair efficiency [33]. Finally, a narrower width of cracks allows for calcium carbonate precipitates to bridge the crack faces more effectively, enhancing the overall mechanical integrity of the repaired region. This bridging effect contributes to a greater structural stability and durability in the repaired areas [34]. 

### 3.4. Morphology and Chemical Compositions

**Figure 7:**
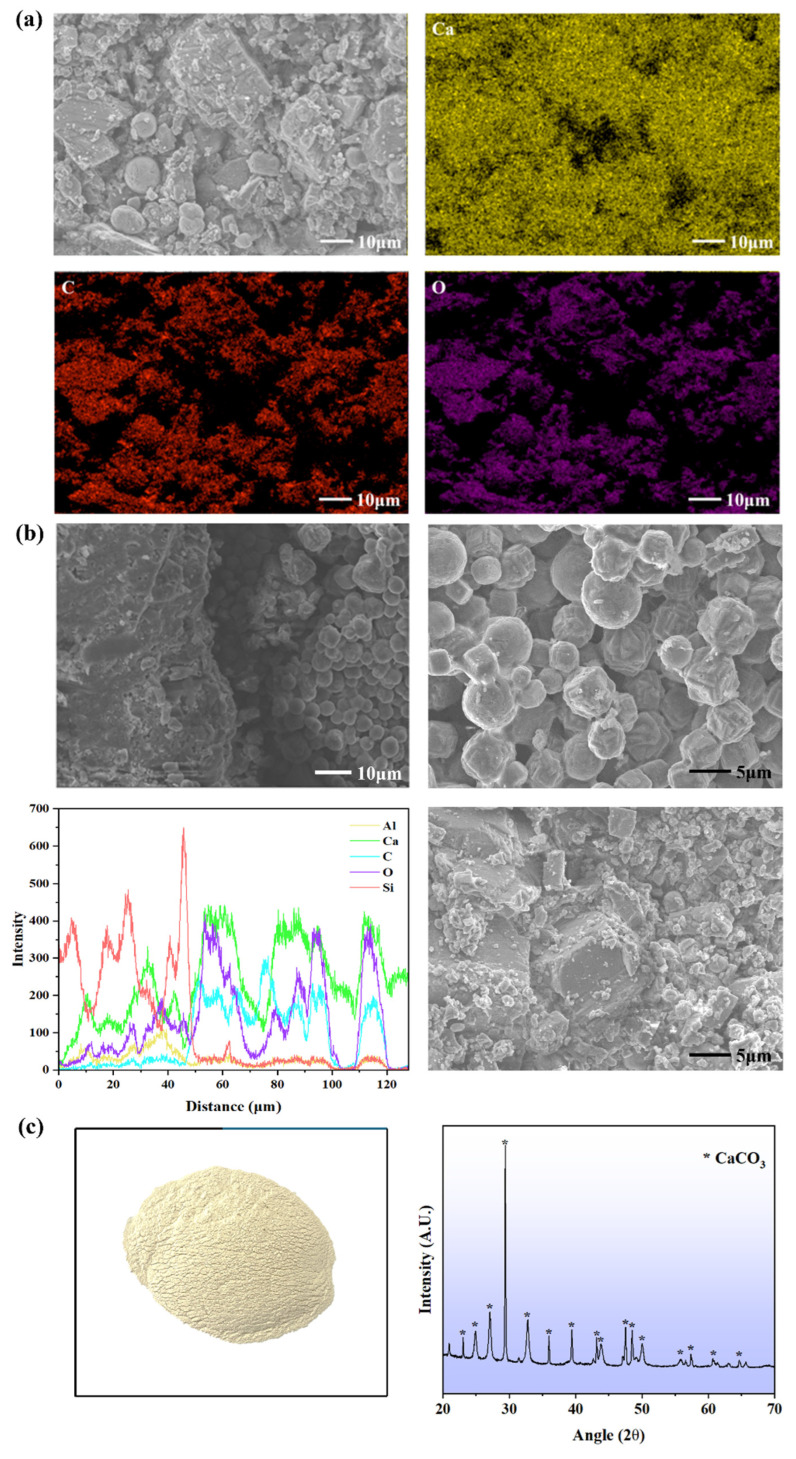
EDS, SEM, and XRD results of mineralization products: (**a**) EDS mapping of mineralization products, (**b**) microstructure and morphology of mineralization products and junction, and EDS line scans of junction, and (**c**) mineralization products and their XRD patterns.

Figure 7 presents the SEM–EDS and XRD images of the repaired products within the cracks. As shown in Figure 7a,b, the rhombohedral- and cubic-shaped minerals, forming massive crystal clusters, were deposited in the cracks in all types of samples. Interestingly, crystals like apricot kernels and spherical spherulites were observed via SEM in some specimens, with sizes ranging approximately from 5 to 20 μm. 

The elemental composition and distribution at the site of repair were further detailed through EDS mapping. The spatial concentrations of essential elements, such as calcium (Ca), carbon (C), and oxygen (O), were highlighted. For instance, the distribution of Ca in the imaged region is shown in Figure 7a. The widespread presence of Ca across the image suggests a high concentration of calcium carbonate (CaCO_3_) deposits, which are typically expected in MICP processes. Darker regions might indicate areas where calcium is less concentrated, possibly corresponding to non-precipitated or partially healed areas. Similarly, the C corresponds to the carbonate ion (CO_3_^2−^) within the calcium carbonate. The distribution of C supports the formation of CaCO_3_, where C aligns with the presence of Ca. Dense red areas suggest an extensive deposition of carbonates, which is a critical indicator of the bio-mineralization mechanism. The O mapping provides insight into the presence of both CaCO_3_ and possibly other oxygen-containing phases like calcium hydroxide (Ca (OH)_2_) or silicate phases from the original cementitious material. The widespread O distribution likely correlates with the CaCO_3_ precipitation.

Figure 7b shows SEM images and ESD line scans for the boundary of a sealed crack and its surrounding cementitious matrix. It is observed that the smooth, rounded precipitates appeared to have grown in clusters, therefore filling gaps within the cementitious matrix. The EDS line scans provide the elemental composition across the boundary between the cementitious material and the bio-mineralized precipitates. For instance, silicon is associated with the cementitious matrix. The peak intensity of silicon is seen more towards the left side (0–50 µm), indicating the cement-based material rich in silicates. Ca peaks at various points across the boundary, with a higher intensity on the right side (around 50–100 µm). This was expected due to Ca being present both in the cement matrix (as part of C-S-H) and in CaCO_3_ precipitation. Similarly, the increased intensity of C in the region between 40 and 120 µm confirms the presence of CaCO_3_ as the primary bio-mineralization product.

An XRD analysis of the white precipitates filling the cracks was also performed to observe their mineralogy. Figure 7c shows the XRD spectra of the biogrout products by *Sporosarcina pasteurii*. The spectral bands suggest the existence of calcite, which concurs with previous findings. A similar study by Wiktor and Jonkers [35] revealed that calcite was the self-healing product by the metabolic activities of *B. alkalinitrilicus* when calcium lactate was added as a calcium source to facilitate bio-mineralization.

## 4. Discussion

In this study, the MICP method, using *Bacillus pasteurii*, was applied to repair concrete cracks of varying dimensions, with the results showing the technique’s promising potential as an alternative to traditional repair methods. 

The aforementioned results indicate that MICP treatment is effective in increasing the UCS of cracked concrete specimens, with the degree of improvement depending on both the width and depth of the cracks. The underlying mechanism is driven by the bio-mineralization process, where CaCO_3_ precipitates into the crack voids, effectively binding the material and restoring some of its original mechanical integrity. In terms of the crack width effect, samples with moderate crack widths (1 mm), particularly B_1_, experienced the most significant strength recovery, which can be attributed to the optimal conditions for microbial CaCO_3_ precipitation. The cracks were large enough to accommodate significant CaCO_3_ precipitation, but not too large for the process to be inefficient. However, narrower cracks (0.5 mm, group A) appeared to have a negligible effect on the mechanical properties, since they did not change much of the integrity of the concrete specimens. On the other hand, for larger cracks (3 mm, group D), the increase in strength was lower, likely because the volume of CaCO_3_ precipitates may not have been sufficient to fully repair the larger voids. The microbial activity might not have been able to bridge the larger gaps effectively. Regarding the effect of crack depth, it also played a significant role in the recovery of compressive strength. In all groups, shallower cracks (10 mm depth) exhibited greater increases in UCS compared to deeper cracks (20 mm and 30 mm). This can be attributed to the better penetration of the microbial solution into shallower cracks, allowing for more effective precipitation of CaCO_3_ throughout the entire crack volume. Deeper cracks, particularly those at a 30 mm depth, are likely less accessible for microbial infiltration, resulting in uneven mineralization and a reduced mechanical repair efficiency. In addition, deep cracks can significantly reduce repair effectiveness due to insufficient oxygen within these cracks. 

The water absorption results highlight the effectiveness of biogrout in reducing the water absorption in cracked concrete, with some improvements in performance observed after bio-mineralization. The significant reduction in the mass change ratio of absorbed water at different crack depths and widths indicates the successful sealing of cracks through MICP. This sealing mechanism can primarily be attributed to the enzymatic precipitation of calcium carbonate within cracks, forming a dense layer that blocks the pathways, to some degree, for water ingress [36,37,38,39]. When the crack width was small (e.g., 0.5 mm or 1 mm), the bio-mineralization process effectively filled the available void space, reducing water transport. However, as the crack width increased to 2 mm or even more, the effectiveness of the sealing process showed a diminished capacity, depending on the crack depth. When the crack depth was fixed at 20 mm, the depth of microbial infiltration appeared to be sufficient to create a barrier to water absorption, thus leading to the observed reductions in mass change ratios. In shallow cracks (10 mm), microbial-induced CaCO_3_ formation likely created an even more effective seal due to the more compacted nature of the precipitate, minimizing water ingress. There was no significant difference in the absorbed water mass change ratio after 48 h, indicating that the concrete pores likely reached saturation equilibrium in the test. Initial absorption was driven by capillary forces and was most prominent in the early hours. Once the capillary forces dissipated due to pore filling, water absorption reached a plateau. In this context, the efficacy of the bio-mineralization process in reducing absorption was most evident in the early phases (such as the first 24 h), where the sealing of cracks by calcium carbonate deposition limited water entry. However, by the 48 h point, both sealed and unsealed cracks exhibited minimal further absorption, indicating that the limiting factor for water ingress was no longer crack geometry, but the saturation of the surrounding material. These results also imply that, in practical applications, the early hours following exposure to water or moisture are critical in assessing the effectiveness of MICP for crack healing. Long-term exposure beyond this initial period may not show further benefits in terms of water absorption reduction, as the concrete has already reached its saturation threshold. Therefore, optimizing MICP treatment for rapid crack sealing and water resistance in the early hours could enhance its practical application in improving the durability of concrete structures exposed to moisture.

In the SEM images, it was found that the morphology of apricot kernels or spherical spherulites appeared, indicating the existence of vaterite [40]. As per Ostwald’s rule, the least stable polymorph generally crystallizes first. Additionally, more stable polymorphs tend to exhibit lower solubility indices. Among the three non-hydrated CaCO_3_ polymorphs, vaterite has the highest solubility index, followed by aragonite and calcite, indicating that calcite is the most stable polymorph, while vaterite is the least thermodynamically stable [41,42]. Factors such as temperature, ion concentration, and pH significantly influence crystal polymorph formation. A previous study with Raman spectroscopy revealed the existence of a spherulitic morphology of vaterite, indicating its prolonged stability as it persisted for several months. Chloride ions seem to facilitate vaterite formation and may play a role in its stabilization [43,44].

## 5. Conclusions

This study presents a comprehensive multi-technique experimental investigation into the efficacy of biogrout in repairing cracked concrete specimens with varying widths and depths, mainly focusing on the enhancement of UCS and water tightness. Based on the aforementioned experimental results, conclusions can be drawn as follows:

- The optimal bacterial activity for CaCO_3_ deposition and, thus, an enhanced repair effectiveness were achieved during the late exponential to early stationary phases (from approximately 24 to 36 h). During this period, the bacterial OD600 increased significantly, demonstrating robust growth.
- MICP treatment was effective in increasing the UCS of cracked concrete specimens, with the degree of improvement depending on both the width and depth of the cracks.
- Specimens with cracks with a 1 mm width and 10 mm depth (group B_1_) showed the most significant increase in UCS, with a maximum improvement of 11.4%**.**
- Based on water absorption tests, it was found that biogrout for concrete crack repair was significantly effective, particularly when the depth and width of the crack were relatively small. In addition, there was no significant difference in the absorbed water mass change ratio when measuring it after 48 h.
- It is suggested that optimizing MICP treatment for rapid crack sealing and water resistance in the early hours could enhance its practical application to improve the durability of concrete structures exposed to moisture.
- SEM–EDS confirmed the presence of CaCO_3_ as the dominant repairing compound. In addition, results from the SEM images indicated that the CaCO_3_ in bacteria-based specimens with calcium chloride showed two polymorphs, namely, calcite and vaterite.

## Figures and Tables

**Table 1:** Microbial medium formulation.

Component	Content
Beef extract	6 g/L
Peptone	10 g/L
Urea	15 g/L
Deionized water	-

**Table 2:** Chemical composition of OPC (%).

Raw Material	SiO_2_	Al_2_O_3_	Fe_2_O_3_	CaO	Na_2_O	K_2_O	MgO	SO_3_	LOI
OPC	21.31	5.32	3.04	63.57	0.14	0.44	1.85	2.49	0.48

**Table 3:** Experimental scheme.

Specimen Number	Crack Width (mm)	Crack Depth (mm)	Crack Length (mm)
A_1_	0.5	10	20
A_2_	0.5	20	20
A_3_	0.5	30	20
B_1_	1	10	20
B_2_	1	20	20
B_3_	1	30	20
C_1_	2	10	20
C_2_	2	20	20
C_3_	2	30	20
D_1_	3	10	20
D_2_	3	20	20
D_3_	3	30	20

**Table 4:** Growth conditions of *Bacillus pasteurii*.

Time (h)	0	4	6	12	24	36	48	54	60
OD600	0.52	0.55	0.64	1.15	1.85	2.31	2.38	2.43	2.45

**Table 5:** Comparison of UCS before and after treatment.

Specimen Number	UCS (MPa)	UCS After Repairing Cracks (MPa)	Increase Ratio of UCS (%)
Crack-free	43.2	-	-
A_1_	37.53	39.42	5.04
A_2_	36.21	37.98	4.89
A_3_	32.27	33.58	4.06
B_1_	33.79	37.65	11.4
B_2_	30.42	33.24	9.27
B_3_	26.98	28.64	6.15
C_1_	32.45	35.66	9.89
C_2_	27.84	30.17	8.37
C_3_	22.71	23.62	4.01
D_1_	29.11	31.43	7.97
D_2_	23.59	25.21	6.87
D_3_	16.54	17.35	4.90

## Data Availability

All data has been shown in this manuscript.
